# Risk factors associated with indicators of dehydration among migrant farmworkers

**DOI:** 10.1016/j.envres.2024.118633

**Published:** 2024-03-09

**Authors:** Chibuzor Abasilim, Lee S. Friedman, Miranda Carver Martin, Dana Madigan, Jose Perez, Maria Morera, Antonio Tovar, Fritz Roka, Nezahuacoyotl Xiuhtecutli, Linda Forst, Paul Monaghan

**Affiliations:** a Division of Environmental and Occupational Health Sciences, School of Public Health, University of Illinois Chicago, Chicago, IL, USA; b Department of Agricultural Education and Communication, University of Florida, Gainesville, FL, USA; c Farmworker Association of Florida, Apopka, FL, USA; d Florida Gulf Coast University, Ft. Myers, FL, USA

**Keywords:** Farmworkers, Dehydration, Heat exposure, Urine specific gravity

## Abstract

**Objective:**

Farmworkers are at increased risk of adverse health outcomes related to occupational heat exposure and inadequate access to water, shade, or rest breaks. Presently, there is a dearth of studies examining the prevalence of dehydration and related factors in U.S. farmworkers. Our objectives were to characterize hydration status during typical workdays and to identify risk factors associated with increased dehydration in migrant farmworkers employed in Florida.

**Methods:**

Urine samples were collected and analyzed for urine specific gravity (USG) 2–3 times per person per day over five days in May 2021 and 2022. Data collection included demographic characteristics, wet-bulb-globe-temperature (WBGT), and information on working conditions (task type, duration, and crop units harvested), fluid intake, clothing worn, and heat safety behaviors. Multivariable mixed regression models were used to evaluate risk factors associated with change in USG levels (continuous) during a work shift.

**Results:**

A total of 111 farmworkers participated in this study providing 1020 cumulative USG measurements, of which 96.8% of end-of-shift USG samples were above 1.020 indicating potential dehydration. In multivariable models, dehydration assessed using change in USG levels significantly declined with age (β = −0.078; 95%CI: 0.150, −0.006) but showed significant increase with body mass index (β = 0.016; 95%CI: 0.003, 0.028), WGBT (β = 0.054; 95%CI:0.044, 0.064), mean shift duration, and state of primary residence. We did not find significant associations of dehydration with type of clothing worn, intake of employer-provided water, or crop units harvested during a shift in this sample of farmworkers.

**Conclusion:**

Our findings underscore the need for additional research to evaluate adverse outcomes related to dehydration and to better understand recovery patterns from chronic dehydration across workweeks and harvest seasons in migrant farmworkers.

## 1. Introduction

Dehydration is one of the primary concerns for persons engaged in strenuous activities under working conditions with elevated temperatures. The evaporation of sweat from the skin surface is one of the body’s key mechanisms for thermoregulation and is essential when metabolic activity is combined with high ambient temperatures (Gauer and Meyers, 2019). The maintenance of adequate fluid levels is central to the prevention of dehydration and heat-related illness (HRI). However, there are no consistent overt signs and symptoms of dehydration until a person is already dehydrated, so evaluating biomarkers can assist in identifying and managing hydration status.

There are multiple accepted ways to measure hydration status, including urine specific gravity (USG), but there is no current gold standard. While USG has some limitations as a marker for hydration status (Cheuvront et al., 2015), it is a well-accepted measure with the following advantages: 1) it is feasible to use in field conditions for both the person providing and the person analyzing the sample; 2) is not cost-prohibitive; 3) it is non-invasive compared to tests that would require samples of blood through fingerstick or venipuncture (Cheuvront et al., 2010; Wagoner et al., 2020; MacPherson et al., 2018); and 4) there is very low intra-person variability across measurements (Cheuvront et al., 2010). USG has been used in numerous studies that demonstrate a relationship between hydration status and outcomes related to heat, stress, and organ function in a range of industries and occupations (Wagoner et al., 2020; Acharya et al., 2018; Ioannou et al., 2022; Mix et al., 2018).

The Occupational Safety and Health Administration (OSHA) recognizes that farmworkers are prone to dehydration and other adverse heat-related health outcomes through exposure to heavy workloads as defined by the American Conference of Governmental Industrial Hygienists (ACGIH) work category classification, warm or hot environments, lack of acclimatization, and clothing that traps body heat (OSHA, 2017; ACGIH, 2017b). While there are well-recognized and effective safety controls to prevent these adverse heat-related health outcomes (Occupational Safety and Health Administration, 2023), outside of the general duty clause, there is no specific federal OSHA standard relating to heat exposure. Currently, California, Minnesota, and Washington are the only states to have laws related to occupational heat exposure (OSHA, 2023). In addition, the agricultural work sector relies heavily on the labor of migrants, primarily from Mexico and Central America, many of whom may not be offered measures to prevent dehydration and other heat-related adverse health effects while meeting the demands of their work (Bethel and Harger, 2014; Fleischer et al., 2013; Bethel et al., 2017; Spector et al., 2015; Chapman et al., 2021).

Farmworkers face unique job tasks and employment conditions that put them at risk of dehydration and heat-related adverse health effects. Only a handful of studies have examined measures of dehydration in farmworkers, and most often in the context of acute kidney injury (Mix et al., 2018; Wesseling et al., 2016; Garcia-Trabanino et al., 2015; Laws et al., 2015, 2016; Moyce et al., 2016, 2017, 2020). Of these studies, the majority were conducted in Central American farmworkers who may share similar characteristics with U.S. migrant farmworkers, but may also differ by access to potable water, working conditions, and underlying health of the workforce (Mix et al., 2018; Wesseling et al., 2016; Garcia-Trabanino et al., 2015; Laws et al., 2015, 2016). Of the prior studies involving seasonal and migrant U.S. farmworkers, reported changes in USG in relation to hydration status have been mixed (increased in Mix et al., 2018; and no change in Spector et al., 2018). In the current study, we expand on prior work to include USG measurements over multiple workdays and include key information relating to work practices in a sample of Florida farmworkers. This study aims to (1) characterize urine specific gravity measures of hydration status across typical workdays in farmworkers in Florida and (2) identify risk factors associated with increased urine specific gravity indicative of dehydration.

## 2. Materials and methods

### 2.1. Farmworker Recruitment

Farmworkers employed at a vegetable farm in south-central Florida were recruited in mid-May of 2021 and 2022 as part of a peer health promoter intervention study funded by the Southeastern Coastal Center for Agricultural Health and Safety. Harvesting is organized by crews comprised of 20–25 members, including a supervisor, loaders, and pickers. We recruited and interviewed 113 farmworkers, but excluded two farmworkers with invalid USG levels (final enrollment: N = 94 in 2021; N = 52 in 2022, of which N = 34 farmworkers had also participated in 2021). The study protocol was approved by the institutional review board at the University of Florida, Gainesville (#201702033) and at the University of Illinois Chicago (#2022–0917). All participants provided verbal informed consent and were compensated with gift cards.

### 2.2. Data collection

Surveys were administered in Spanish by trained, fluent Spanish speakers. Baseline survey data were collected before on-site sampling began, and shift-specific daily surveys were collected over three and two days in 2021 and 2022, respectively. Baseline surveys assessed demographic information, body mass index (BMI), work history, and heat safety attitudes and behaviors. Daily surveys were also administered at the end of each workday to gather information on daily working conditions, fluid intake, clothing worn, and heat safety behaviors. Productivity data were also collected from supervisors, which included task type, start and stop times, and units of each crop harvested by a farmworker when working as a picker. Our research team inspected and validated 15–20% of collated data for duplicates and errors to ensure data quality.

### 2.3. Urine specific gravity

Urine specific gravity (USG) was measured by analyzing urine samples with a digital refractometer (Reichert TS Meter-D, Depew, NY). The reading range of the refractometer is 1.000–1.060, with a precision of 0.0001. The normal range of USG is between 1.003 and 1.035, representing the ratio of the weight of urine to the weight of an equal volume of water. For morning samples (2021 and 2022), workers were provided sterile collection cups the day before and instructed to bring a sample of their first urine of the day to work the following day. The mid-shift samples (2021) and the end-of-shift samples (2021 and 2022) were collected when crews returned to the central packinghouse at lunch (2021) and at the end of their workday (2021 and 2022). While not a true 24-h urine collection, this sampling method has been shown to approximate 24-h urine collection (Perrier et al., 2013a).

For all urine collections, workers were provided sterile closed plastic sample cups in brown paper bags and returned them in the same bags. Samples were stored in coolers until the refractometer analysis. Samples were analyzed on the same day they were collected. The refractometer was calibrated with distilled water at the beginning of each analysis session to account for differences in ambient lighting across collection points. Sample measurements were taken by pipetting approximately 3 drops of urine into the sample well so that the prism surface was covered. After the digital reading was recorded, a cotton ball was used to absorb the sample, the sample well was rinsed with distilled water, and another cotton ball was used to absorb the distilled water.

### 2.4. Weather data

Wet bulb globe temperature (WBGT) and heat index were calculated using air temperature, relative humidity, wind speed, solar irradiance, time of day, and geolocation of the farm (Liljegren et al., 2008; NOAA, 2023; Grundstein et al., 2015). Weather data were downloaded from the Florida Automated Weather Network (FAWN), which provides measures at 15-min intervals (Florida Automated Weather Network, 2023). The values were averaged between the two closest FAWN stations to the farm which are 30 miles apart. The farm was located approximately equidistant between the two weather stations. We present temperature data in both degrees Celsius and Fahrenheit. For reference, we also use the WBGT threat levels for the southeastern U.S. (25.7 °C/78.3°F; Grundstein et al., 2015), locally defined extreme conditions in the 90th percentile warm season (May–September) WBGT, and existing American College of Sports Medicine (ACSM) thresholds.

### 2.5. Statistical analysis

For the descriptive analysis, we provide mean and distribution of USG levels at the start and end of shifts stratified by farmworker demographics, health attributes, health behaviors, clothing worn during a shift, common symptoms associated with heat-related illness or dehydration, work activity, consumption of employer-provided water during a shift, and wet bulb globe temperature. We use the CDC-NIOSH guidelines for fluid consumption under hard working conditions specific to the WBGT reported by the weather stations (24–32 fluid ounces per hour; National Institute of Occupational Safety and Health et al., 2016). For shift-specific variables, we use the parameter of person-shifts to measure each unique farmworker once per shift; if a farmworker worked all five shifts there would be a total of five person-shifts representing that farmworker. Assessment of the normal probability plot, kurtosis, and skewness values indicated that the USG levels were normally distributed (mean = 102.5; median = 102.6; skewness = −0.29; kurtosis = −0.12; Shapiro-Wilkes Test Normality, W = 0.99), so we did not transform the variable. For descriptive statistics, our study used three different thresholds of USG to assess dehydration. We evaluated three cutoffs, namely the primary cut-off of >1.020 in USG (Sawka et al., 2007; Cheuvront et al., 2010), a second cut-off of >1.025 used by the National Collegiate Athletics Association (NCAA; Baron et al., 2015), and a third empirically derived cut-off of >1.028 based on military personnel (Cheuvront et al., 2010). Our final analysis utilized the primary cut-off and presented results based on this cutoff.

For the multivariable analysis, we developed two mixed regression models to control for within-farmworker variance related to repeated USG measurements during each work shift and evaluated factors associated with the change in USG levels (continuous variable) during a work shift. Mixed models can accommodate unbalanced data patterns with the assumption that the missingness is independent of unobserved measurements but dependent on the observed measurements. The absence of mid-shift USG measures from 2022 is expected to have no effect on the estimates. We evaluated various covariance structures based on a priori knowledge and model fit statistics (AIC, log-likelihood) to determine the appropriate random effects component of the model (Kincaid, 2006). For the final models, we utilized a non-stationary first-order autoregressive covariance structure (heterogeneous ARH (1)). In both final models, the time of USG measurements (morning, mid-shift, and end-of-shift for 2021; and morning and end-of-shift for 2022) was included as a random effect. We selected variables to include as fixed effects in final models based on a priori subject matter knowledge from the literature along with statistical evaluation of covariates (standard errors, statistical significance, collinearity) and change in model fit statistics (AIC, log-likelihood; Walter and Tiemeier, 2009). The medical literature reports a strong association between USG and BMI (Macpheson, 2018; KuiperO’Brien et al., 2021), age (KuiperO’Brien et al., 2021), WBGT (Wagoner et al., 2020), fluid intake, workload, and clothing (Mix et al., 2018; Wesseling et al., 2016; Garcia-Trabanino et al., 2015) which can directly impact heat exchange and fluid loss. To improve model estimation, we converted USG values to factor G defined as (SG-1.000)×100.

The parameter estimates represent the unmarginalized change in clinical USG. Because some of the variables had a higher number of missing values, we developed two multivariable models in order to evaluate the stability of the parameter estimates. In model 1, we only included variables with a low number of missing values and omitted potential confounders; only 47 out of 1020 observations were listwise deleted. In model 2, we included body mass index and important potential confounders known to impact body heat exchange and USG (hydration, clothing, and type of work); 198 out of 1020 observations were listwise deleted in this model because of missing values. A two-sided p-value less than 0.05 was considered statistically significant.

## 3. Results

A total of 111 farmworkers participated in this study providing a total of 1020 cumulative USG measurements. The farmworkers worked 370 person-shifts across the five days of data collection in 2021 and 2022. Table 1 presents the distribution of farmworker USG levels at the start and end of shifts by demographic, health behavior, and general health characteristics. Nearly all the farmworkers were male (n = 106; 95.5%), 35 years or younger (n = 92; 82.9%), and most had completed more than a primary education (n = 73; 65.8%). Almost all the farmworkers had migrated from Mexico to work (n = 108; 97.3%) with 2.7% (n = 3) arriving from Guatemala. While many have been working in agriculture for 11 or more years (57.7%; Table 1), most have been working in the U.S. for 5 years or less; 40.5% had been working in the U. S. for less than 1 year (n = 45) and 43.2% for 1–5 years (n = 48) (not shown in tables). Table 2 presents the distribution of WBGT and heat index for each day of observation during the month of May. While both WBGT and heat index increased from the start to the end of each shift, WBGT measurements did not exceed the low threat level for the southeastern U.S. [25.7 °C (78.3 °F)] except on one day – May 17, 2022 (Table 2).

Table 3 presents the number of person-shifts by work characteristics. The median shift duration across all the days of observation was 9.5 h; however, 17.2% of person-shifts were 10–12.9 h long, and 30.6% were 13 h or longer. The weighted average number of hours worked during the previous week was 51.5 h; however, 59.1% of farmworkers reported working a weighted average of 61.2 h during the previous week. The average workweek was 6 days long, with Sundays off, and during each shift, farmworkers were provided a single 30-min break for lunch (not shown in Table 3). If the farmworker was tasked with harvesting crops, they were paid a piece rate which varied for each crop, with the minimum wage as the base salary when the piece rate did not exceed the minimum wage.

Across the shifts, farmworkers self-reported wearing light-colored clothing in 64.6% of the person-shifts, avoided wearing tight clothing in 44.1% of person-shifts, and wore a hat in 50.6% of person-shifts. The primary work activities observed during shifts were harvesting crops, loading/carrying bins filled with the crops harvested by other workers, laying protective plastic covering, or participating in training. In 337 person-shifts (91.1%), the farmworker engaged in harvesting at least one crop. Most of the workers specialized in harvesting, while about two workers in each crew specialized in loading. Person-shifts were therefore primarily divided into two groups: (1) harvesting (81.9% of person-shifts) with a few hours of training or laying plastic, and (2) loading/carrying crops (6.8% of person-shifts) with a few hours of training or laying plastic.

In compliance with the OSHA field sanitation standard, farmworkers were provided 4-ounce cups to use for fluid intake during shifts, and workers could supplement fluid intake with their own personal supply of fluids. Based on fluid intake from owner-operator supplied water (4 oz. cups), only 18.4% of person-shifts had a fluid intake of 24 ounces or greater per hour (CDC recommended hydration level), with 55.3% of person-shifts with less than 12 ounces of fluid intake per hour (not shown in Table 3). A truck with the employer-supplied water cooler towed two portable toilets and was parked at the edge of the field during each shift. When farmworkers switched tasks/crops, the crew loaded into vehicles and had a brief period to rest and rehydrate while moving to the next field. Time spent on each harvest task varied widely, from 15 min to over 8 h. Working multiple fields during the course of a single day was common; 295 person-shifts involved working three or more crops.

Mean levels and distribution of USG by farmworker demographics and working conditions are presented in Tables 1 and 3 The mean USG level at the start of shifts was 1.022 (sd = 0.005) and was 1.029 (sd = 0.004) at the end of shifts. In both 2021 and 2022, sampling began on Mondays. Of the 1020 USG samples taken, 826 (81.0%) had USG levels higher than 1.020, indicating possible dehydration, of which 62.1% of start-of-shift samples and 96.8% of end-of-shift samples were above 1.020. Based on the higher NCAA cut-off for dehydration, 25.1% of start-of-shift samples and 82.7% of end-of-shift samples were above 1.025. Using a cut-off based on military personnel, 11.5% of start-of-shift samples and 55.4% of end-of-shift samples were above 1.028. A total of 24 out of 1020 USG measurements, involving 17 farmworkers, were above the higher upper range of 1.035. Table 2 shows the mean USG levels for each day and across shifts. While start-of-shift USG levels are consistently lower than end-of-shift USG levels each day, the morning USG increases by an average of 0.002 across each consecutive day. USG levels increased by an average of 0.007 from start to end of shifts across all person-shifts.

Based on the fully adjusted multivariable model (model 2), USG levels declined with age (Table 4). In contrast, USG levels were significantly higher among farmworkers who migrated from the states of Chiapas, Oaxaca, or Guerrero in Mexico (0.002 higher; p = 0.002). Each 5-point increase in BMI was associated with an increase in USG by 0.001 (p = 0.014). On average, each shift showed an increase of 6.1 °C (10.9 °F) in WBGT which is associated with an increase of 0.006 in USG (p <0.001). The mean shift duration was 10.2 h which is associated with a 0.001 increase in USG (p = 0.003). In this sample, type of clothing, harvesting crops, fluid intake of employer-provided water, piece rate, number of crops worked during a shift, or symptoms associated with dehydration were not significantly associated with USG levels.

## 4. Discussion

Research in Florida has documented that farmworkers are routinely exposed to extreme heat and humidity while harvesting raw produce (Mac et al., 2017; Mix et al., 2018). Our study population of farmworkers employed on a south-central Florida vegetable farm presented with chronically high USG levels above 1.020. Our models show that the largest change in daily USG levels was associated with increasing WBGT, even though most workdays did not have peak WBGT measurements exceeding the low threat level in Florida (Grundstein et al., 2015). However, both NIOSH and ACGIH recommend adjusting the WBGT limit downwards to 25 °C (77 °F) for acclimated workers with heavy workloads and a further downward adjustment of 3 °C–22 °C (71.6 °F) for workers wearing double-layer clothing (clothing adjustment factor), which is common among these farmworkers (ACGIH, 2017). With the downward adjustment, all days of observation had WBGT levels well above the adjusted threat level. Independent of WBGT, total hours worked during a shift, BMI, lower age in years, and home state in Mexico were also associated with elevated USG levels.

In this population, 96.8% of end-of-shift USG samples were above 1.020, substantially higher than levels reported in other outdoor workers with heavy workloads (Bates et al., 2010; Biggs et al., 2011; Orysiak et al., 2022), but similar to another report involving Florida farmworkers (Mix et al., 2018). Among these Florida farmworkers, USG levels increased by an average of 0.007 during a shift. By comparison, studies of healthy adults show that daily USG levels fluctuate on average between 0.002 and 0.003, with a daily fluctuation of 0.003 in low fluid drinkers (Perrier et al., 2013b, 2017; Athanasatou et al., 2019). In a comparable analysis of Florida farmworkers, the average daily change in USG was 0.004 (Mix et al., 2018). In a study of young adults in the military who were euhydrated at the start of each day, an intra-day change of 0.007 in USG was associated with a 80–90% likelihood of dehydration (Cheuvront et al., 2010). While USG levels decreased between the end of a shift and the following morning, Tuesday and Wednesday USG levels did not return to baseline morning levels observed at the start of the workweek (Monday). There appears to be a longitudinal increase in USG levels across sequential workdays by an average of 0.002, indicating possible progressive dehydration (Baron et al., 2015). Furthermore, 62.1% of start-of-shift USG samples were >1.020, which may be further evidence of chronic progressive dehydration (Baron et al., 2015; Kavouras, 2002; Sawka et al., 2007). Because these farmworkers do not have baseline USG measurements taken at the start of the harvest season and were not followed across multiple sequential workweeks, we do not know if the farmworkers experienced recovery in USG levels on days off or between harvest seasons.

Importantly, the working conditions of farmworkers do not support euhydration even when the employer is in compliance with the OSHA field sanitation standard. The standard conditions of the industry incentivize working faster and longer, with minimal breaks and stops for water, all of which are associated with elevated USG levels (Baron et al., 2015). The working conditions are characterized by (1) very long hours usually for six days a week, (2) no mandatory rest breaks, (3) financial compensation based on a piece rate without overtime compensation, (4) risk of losing a shift or not being rehired in the next season, (5) an environment in which adequate hydration of 24–32 oz./hour is time-consuming because of small cups and slow fluid dispensers, and (6) vulnerability related to immigration status, whether workers are undocumented or documented as ‘guest workers’ under the H-2A program (Arcury and Mora, 2020; Onel et al., 2020). These conditions combine to disincentivize HRI-preventive behaviors if workers fear that the time it takes to rest, rehydrate, and urinate could impact their piece rate-based wages. The cornerstone of safety recommendations to limit work-related heat injury, dehydration, and adverse health effects caused by strenuous physical exertion is to ensure that workers have time to acclimate to heat and have access to rest, ventilation/air flow, shade, and water (National Institute of Occupational Safety and Health et al., 2016). However, there is an absence of regulatory protections for US farmworkers, who (1) are expressly exempted from segments of the Fair Labor Standards Act (FLSA) that require overtime pay (US Department of Labor, 2023), (2) Florida does not have a state law requiring mandatory rest breaks regardless of shift duration, and (3) there is no current heat standard in the Occupational Safety and Health Act. The OSH Act sanitation standard is the only standard that specifically requires potable water and toilet access (OSHA, 2023).

Dehydration is not synonymous with heat exposure but can amplify the negative effects of a heavy workload, making workers more susceptible to fatigue and heat-related illnesses. Dehydration can acutely impact work productivity as it is associated with muscle weakness, fatigue, lower endurance, headaches, muscle cramps, lightheadedness, dizziness, and reduced cardiac function (Sawka et al., 2007; Murray, 2007; Baron et al., 2015); it also increases the risk of heat-related illness because of impaired internal thermoregulation (Baron et al., 2015). Prolonged extreme dehydration and excessive strenuous workload are also associated with chronic diseases: increased risk of renal dysfunction and nephrolithiasis, and, possibly, chronic kidney disease of unknown etiology (CKDu) mostly observed in agricultural communities, rhabdomyolysis, asthma, cardiovascular disease, stroke, and conditions related to oxidative stress (Garcia-Trabanino et al., 2015; Wesseling et al., 2016; Paik et al., 2009; Popkin et al., 2010; Strippoli et al., 2011; Clark et al., 2011; Baron et al., 2015). To fully address the issue of acute and chronic health impacts of dehydration and HRI among farmworkers, regulatory changes must address living wages and safe working conditions for hired farmworkers along with financial safeguards or cost offsets for owner-operators to sustainably provide them. In the absence of these larger-scale changes, there are practical engineering and administrative controls that can negligibly impact the income of farmworkers and owner-operators, while simultaneously addressing the acute and long-term health risks of farmworkers. At a minimum, these include providing (1) easily accessible and rapidly dispensed fluids (including personal fluid dispensers while working in the field, similar to those used in the military), (2) proximal clean urination facilities (within 100–200 ft of workers), (3) personal protective clothing provided at no cost to workers that provides skin protection and good heat exchange, (4) mobile fans to increase airflow when ambient air temperatures exceed skin temperatures, and (5) mobile shade units which are associated with both heightened perceived comfort and reductions in globe temperatures of 4–7 °C (Middel et al., 2016; National Institute of Occupational Safety and Health et al., 2016).

There are several limitations to this analysis. First, data on diabetes, renal disease, other comorbidities, and diet (e.g. sodium, sugar consumption), all of which impact USG levels, are lacking in this study. Second, no data was collected on other important blood and urine-based parameters of dehydration and related adverse health outcomes, including plasma osmolality, change in percent body weight, blood urea nitrogen (BUN) to creatinine ratio, urine sodium concentration, protein, blood, leukocytes, glucose, pH, bilirubin, ketone, nitrite, and urobilinogen. Studies have reported that test subjects may have a dehydration-level USG with a normal serum sodium level (Hooper et al., 2016), as a result of the kidney’s role in conserving water and maintaining normal serum sodium levels (which increases USG levels). However, the farmworkers in this study have chronically elevated USG, even at the start of shifts. Furthermore, USG has been shown to be a superior marker of chronic dehydration than plasma osmolarity because of kidney adaptation, which over time does a better job stabilizing plasma osmolarity in the chronically dehydrated (Perrier et al., 2013a,b; Pross et al., 2013; Baron et al., 2015). Third, USG may be biased by atypical specimen collection; morning urine samples were provided by farmworkers, not under the auspices of the research team, and mid-day and post shift samples were collected in the field, rather than a lab setting. However, research indicates a negligible inter-subject variability in USG (Cheuvront et al., 2010). In addition, a refractometer was used for all USG measurements, which has been shown to provide significantly lower USG measurements compared to hydrometers or reagent strips (Stuempfle et al., 2003). Fourth, both daily clothing and fluid consumption were self-reported. Employer-provided fluid consumption could not be validated, and the complexity of measuring personal beverage use made controlling for supplementary fluids impractical. In addition, our research team observed incongruence between self-reported and observed clothing worn during a shift. These factors might explain the absence of an observed association in our analysis between USG levels and clothing and employer-provided fluid intake.

## 5. Conclusions

Without fundamental changes to regulatory protections that address wages, hours, and workplace safety, in conjunction with labor and price safeguards, workplace interventions to improve hydration in the interim should focus on collaborating with employers to implement engineering and administrative controls. OSHA has proposed a new heat standard that would cover farmworkers working in larger operations, but the proposed rule is still under review (Occupational Safety and Health Administration, 2021). While it is unclear if our findings are generalizable to the wider US hired farmworker workforce, the findings of this study indicate that farmworkers may be chronically dehydrated. There is a need for research to more accurately assess the frequency of dehydration among farmworkers in the United States throughout workweeks and across different harvest seasons. Additionally, it is essential to investigate the interaction effects of extreme heat on dehydration in the context of heavy workload. This information will contribute to the development of policies and interventions addressing dehydration from extreme heat stress in the farmworker population.

## Figures and Tables

**Table 1:** Urinary specific gravity levels at the start and end of each shift by demographic characteristics among hired south-central Florida farmworkers, 2021 and 2022

	Number of Farmworkers^a^	Urinary Specific Gravity at Start of Shift				Urinary Specific Gravity at End of Shift			
		Mean	sd	min	max	Mean	sd	min	max

**Year**									
2021	93	1.022	0.005	1.009	1.040	1.029	0.004	1.018	1.041
2022	52^a^	1.022	0.005	1.010	1.039	1.028	0.005	1.014	1.037
**Gender**									
Male	106	1.022	0.005	1.009	1.040	1.029	0.004	1.014	1.041
Female	1	1.021	0.003	1.017	1.024	1.030	0.003	1.027	1.033
Unspecified/Other	4	1.023	0.004	1.018	1.028	1.029	0.004	1.022	1.036
**Age**									
Under 26 years	42	1.022	0.005	1.009	1.039	1.030	0.004	1.020	1.037
26–35 years	50	1.022	0.006	1.010	1.040	1.029	0.004	1.014	1.041
36–45 years	16	1.023	0.005	1.011	1.033	1.028	0.004	1.018	1.037
46 years or older	1	1.022	0.005	1.013	1.029	1.025	0.005	1.018	1.036
Unspecified	2	1.021	0.004	1.019	1.023	1.027	0.004	1.022	1.032
**Home Residence**									
Guanajuato, Mexico	31	1.022	0.006	1.010	1.039	1.028	0.004	1.014	1.037
Veracruz, Mexico	4	1.023	0.005	1.017	1.032	1.028	0.003	1.022	1.032
Chiapas, Mexico	9	1.022	0.005	1.013	1.034	1.029	0.004	1.020	1.036
Oaxaca, Mexico	8	1.025	0.005	1.015	1.034	1.031	0.003	1.026	1.040
Michoacan, Mexico	15	1.021	0.006	1.009	1.036	1.029	0.004	1.020	1.037
Guerrero, Mexico	13	1.024	0.005	1.012	1.033	1.031	0.003	1.025	1.036
Baja California, Mexico	15	1.023	0.005	1.012	1.040	1.029	0.004	1.016	1.041
Other State in Mexico	13	1.021	0.005	1.013	1.030	1.028	0.004	1.018	1.033
Guatemala	3	1.022	0.005	1.011	1.028	1.028	0.004	1.020	1.035
**Education**									
Primary School	38	1.023	0.005	1.011	1.034	1.029	0.004	1.016	1.036
High School	62	1.022	0.006	1.009	1.040	1.029	0.004	1.014	1.041
Preparatory School	6	1.021	0.004	1.013	1.031	1.028	0.004	1.020	1.035
Unspecified 5		1.023	0.004	1.015	1.028	1.030	0.004	1.018	1.037
**Total Years Worked in Agriculture**									
Less than 1 year	2	1.024	0.002	1.021	1.025	1.029	0.003	1.027	1.033
1–5 years	22	1.022	0.005	1.009	1.032	1.029	0.004	1.014	1.037
6–10 years	22	1.022	0.006	1.010	1.039	1.029	0.004	1.014	1.040
11 –15 years	38	1.022	0.005	1.011	1.040	1.029	0.004	1.018	1.041
16–20 years	11	1.023	0.006	1.011	1.032	1.030	0.004	1.022	1.036
More than 20 years	15	1.023	0.005	1.011	1.034	1.028	0.004	1.018	1.036
Unspecified	1	1.021	0.004	1.013	1.027	1.029	0.004	1.023	1.034
**BMI**									
Less than 18.5	0	~	~	~	~	~	~	~	~
18.5 to 24.9	25	1.021	0.005	1.009	1.034	1.028	0.004	1.018	1.040
25 to 29.9	45	1.022	0.006	1.010	1.037	1.029	0.004	1.018	1.036
30 or higher	32	1.023	0.005	1.012	1.040	1.030	0.004	1.018	1.041
Missing	9	1.022	0.005	1.011	1.032	1.028	0.005	1.014	1.035
**Wellbeing during shift**									
Very Bad	5	1.027	0.004	1.022	1.030	1.030	0.003	1.027	1.033
Bad	3	1.024	0.007	1.013	1.039	1.030	0.003	1.026	1.033
Neither Bad nor Good	21	1.023	0.006	1.010	1.036	1.030	0.004	1.020	1.040
Good	64	1.022	0.005	1.009	1.040	1.029	0.004	1.014	1.041
Very Good	18	1.022	0.005	1.012	1.034	1.029	0.004	1.018	1.036

a For counts by year, the numbers are not independent; In 2022, N = 18 new farmworkers participated in 2022 with an additional N = 34 farmworkers from 2021 who participated again in 2022.

**Table 2:** Wet bulb globe temperature and heat index at the start, middle, and end of shift on each sampling date among hired south-central Florida farmworkers, 2021 and 2022.

Date	Day of Week	Time of Shift	Wet Bulb Globe Temperature in Degrees Celsius (Fahrenheit)	Heat Index in Degrees Celsius (Fahrenheit)	Mean USG (sd)

5/17/2021	Monday	Start	17.6 (63.7)	20.0 (68.0)	1.020 (sd = 0.004)
5/17/2021	Monday	Middle	21.0 (69.8)	23.7 (74.7)	1.024 (sd = 0.004)
5/17/2021	Monday	End	23.3 (74.0)	27.9 (82.2)	1.029 (sd = 0.004)
5/18/2021	Tuesday	Start	18.1 (64.6)	20.0 (68.0)	1.022 (sd = 0.005)
5/18/2021	Tuesday	Middle	21.9 (71.4)	24.7 (76.4)	1.024 (sd = 0.005)
5/18/2021	Tuesday	End	24.0 (75.2)	28.8 (83.9)	1.029 (sd = 0.004)
5/19/2021	Wednesday	Start	19.7 (67.4)	21.4 (70.5)	1.024 (sd = 0.006)
5/19/2021	Wednesday	Middle	22.5 (72.6)	25.1 (77.1)	1.027 (sd = 0.005)
5/19/2021	Wednesday	End	24.2 (75.5)	28.8 (83.8)	1.031 (sd = 0.004)
5/16/2022	Monday	Start	19.5 (67.0)	17.0 (62.6)	1.021 (sd = 0.006)
5/16/2022	Monday	End	25.6 (78.0)	30.5 (87.0)	1.026 (sd = 0.005)
5/17/2022	Tuesday	Start	18.7 (65.6)	16.5 (61.6)	1.023 (sd = 0.005)
5/17/2022	Tuesday	End	26.6 (79.9)	31.4 (88.6)	1.030 (sd = 0.003)

**Table 3:** Urine specific gravity levels at the start and end of each shift by type of clothing worn, symptoms during shift, and work load among hired south-central Florida Farmworkers, 2021 and 2022.

	Urine Specific Gravity at Start of Shift					Urine Specific Gravity at End of Shift				
			
	Person-Shifts	Mean	sd	min	max	Mean	sd	min	max	Percent Above USG 1.020 at end of Shift

**Total Hours Worked During Shift**										
Less than 8 Hours	80	1.023	0.006	1.012	1.039	1.029	0.005	1.014	1.040	93.8%
8–10 h	96	1.022	0.006	1.011	1.036	1.030	0.003	1.020	1.041	99.0%
10–13 h	58	1.021	0.005	1.011	1.031	1.028	0.004	1.014	1.035	96.6%
13 or More hours	103	1.023	0.005	1.009	1.040	1.029	0.004	1.020	1.037	97.1%
**Work Activity**										
Picking herbs/vegs	303	1.022	0.005	1.009	1.040	1.029	0.004	1.014	1.041	97.0%
Loader/Carrier	25	1.022	0.006	1.011	1.032	1.028	0.005	1.014	1.035	92.0%
Laying plastic or participated in training	97	1.022	0.004	1.012	1.033	1.030	0.004	1.022	1.036	100.0%
**Wore Light Colored Clothing During Shift**										
No	126	1.022	0.005	1.009	1.034	1.029	0.004	1.018	1.040	96.8%
Yes	230	1.022	0.006	1.010	1.040	1.029	0.004	1.014	1.041	96.5%
**Avoided Wearing Tight Clothing During Shift**										
No	199	1.022	0.005	1.010	1.036	1.029	0.004	1.014	1.040	96.5%
Yes	157	1.022	0.006	1.009	1.040	1.029	0.004	1.014	1.041	96.8%
**Wore Hat During Shift**										
No	176	1.022	0.006	1.009	1.037	1.029	0.004	1.018	1.041	97.2%
Yes	180	1.022	0.005	1.011	1.040	1.028	0.004	1.014	1.037	96.1%
**Total Different Crops Worked During Shift**										
1 or 2 Crops	42	1.023	0.007	1.009	1.040	1.027	0.005	1.014	1.036	85.7%
3 Crops	135	1.023	0.005	1.011	1.034	1.030	0.004	1.014	1.041	97.8%
4 Crops	48	1.022	0.004	1.013	1.030	1.029	0.003	1.022	1.036	100.0%
5 or more Crops	112	1.022	0.005	1.010	1.037	1.029	0.004	1.018	1.040	98.2%
**Employer Provided Water**										
Less than 4 ounces per hour	74	1.022	0.006	1.011	1.039	1.028	0.004	1.014	1.037	97.3%
4–8oz	59	1.022	0.005	1.010	1.034	1.029	0.004	1.018	1.035	96.6%
8–16oz	74	1.023	0.005	1.013	1.034	1.030	0.004	1.014	1.036	98.7%
16–24 oz per hour	45	1.023	0.005	1.012	1.037	1.030	0.004	1.020	1.040	97.8%
24 or More oz per hour 57		1.021	0.006	1.012	1.040	1.029	0.004	1.018	1.041	96.5%
**Persons Reporting Symptoms During Work Shifts**										
No reported symptoms	302	1.022	0.005	1.009	1.040	1.029	0.004	1.014	1.041	97.0%
Headache	42	1.022	0.005	1.013	1.034	1.028	0.005	1.014	1.040	95.2%
Cramp	28	1.024	0.005	1.014	1.036	1.029	0.004	1.020	1.037	96.4%
Dizziness	6	1.019	0.004	1.013	1.023	1.027	0.002	1.024	1.030	100.0%
Nausea or Vomiting	7	1.023	0.003	1.018	1.028	1.030	0.004	1.025	1.035	100.0%

**Table 4:** Multivariable mixed regression models evaluating personal factors and working conditions associated with urine specific gravity levels among hired southcentral Florida Farmworkers, 2021 and 2022.

	Model 1^a^		Model 2^b^	
	Beta (95% CI)^c^	P-value	Beta (95% CI)^c^	P-value

**Demographics**				
Age (ordinal)	−0.086 (−0.156, −0.016)	0.0168	−0.078 (−0.150, −0.006)	0.034
Migrated from the States of Chiapas, Oaxaca, or Guerrero in Mexico	0.194 (0.083, 0.305)	0.0007	0.170 (0.045, 0.294)	0.008
BMI	~	~	0.016 (0.003, 0.028)	0.014
**Work Factors**				
Wet Bulb Globe Temperature (per degree Fahrenheit)	0.047 (0.038, 0.056)	<0.001	0.054 (0.044, 0.064)	<0.001
Total Hours Worked During Shift Minus Any Time Absent	0.017 (0.008, 0.025)	<0.001	0.014 (0.005, 0.023)	0.003
**Covariates**				
Worked as a Harvester During Shift	0.092 (−0.013, 0.196)	0.085	0.075 (−0.047, 0.196)	0.228
Insufficient Fluid Intake^d^	~	~	0.009 (−0.083, 0.101)	0.846
Wore Light Colored Clothing During Shift	~	~	−0.004 (−0.080, 0.073)	0.927
Avoided Wearing Tight Clothing During Shift	~	~	−0.003 (−0.083, 0.077)	0.939
Wore Hat During Shift	~	~	−0.012 (−0.093, 0.068)	0.762
**AIC**	**1203.9**		**1028.7**	
**Total N Missing in Model (out of 1020 observations)**	**47**		**198**	

a Model 1: Model omits variables with high missingness. Mixed regression model time of urinary specific gravity measurement as a random effect, using a autoregressive heterogenous (1) variance-covariance matrix.

b Model 2: Model includes variables with high missingness including potential confounders known to impact body heat exchange and USG (hydration, clothing and type of work). Mixed regression model time of urinary specific gravity measurement as a random effect, using a autoregressive heterogenous (1) variance-covariance matrix.

c Urinary Specific Gravity levels used in the model are based on G factor (multiplied by 100). To calculate the actual USG change, the parameter estimates need to be divided by 100.

d All workers who drank less than 24oz. of employer-provided fluid per hour during shift based on CDC recommendation to consume more than 24 ounces of fluid per hour when engaged in strenuous outdoor work.

## Data Availability

The authors do not have permission to share data.
